# Development of a particle swarm optimization-backpropagation artificial neural network model and effects of age and gender on pharmacokinetics study of omeprazole enteric-coated tablets in Chinese population

**DOI:** 10.1186/s40360-022-00594-2

**Published:** 2022-07-19

**Authors:** Yichao Xu, Jinliang Chen, Dandan Yang, Yin Hu, Bo Jiang, Zourong Ruan, Honggang Lou

**Affiliations:** Center of Clinical Pharmacology, School of Medicine, the Second Affiliated Hospital of Zhejiang University, Hangzhou, Zhejiang China

**Keywords:** Plasma concentration, Pharmacokinetics study, Chinese population, Omeprazole, PSO-BPANN

## Abstract

**Background:**

The effects of age and gender were explored on pharmacokinetics study of omeprazole enteric-coated tablets in Chinese population and a plasma concentration prediction model was developed. All the data (demographic characteristics and results of clinical laboratory tests) were collected from healthy Chinese subjects in pharmacokinetics study using 20 mg omeprazole enteric-coated tablets. A noncompartmental method was used to calculate pharmacokinetic parameters, and 47 subjects were divided into two groups based on the calculation of the median age. Pharmacokinetic data from the low-age and high-age groups or male and female groups were compared by Student t-test. After a total of 12 variables were reconstruct and convert into independent or irrelative variables by principal component analysis, particle swarm optimization (PSO) was used to construct a backpropagation artificial neural network (BPANN) model.

**Result:**

The model was fully validated and used to predict the plasma concentration in Chinese population. It was noticed that the C_max_, AUC_0-t_, AUC_0-∞_ and t_1/2_ values have significant differences when omeprazole was administered by low-age groups or high-age groups while there were slight or no significant differences of pharmacokinetic data were found between male and female subjects. The PSO-BPANN model was fully validated and there was 0.000355 for MSE, 0.000133 for the magnitude of the gradient, 50 for the number of validation checks. The correlation coefficient of training, validation, test groups were 0.949, 0.903 and 0.874.

**Conclusion:**

It is necessary to pay attention to the age and gender effects on omeprazole and PSO-BPANN model could be used to predict omeprazole concentration in Chinese subjects to minimize the associated morbidity and mortality with peptic ulcer.

**Trial registration:**

The study was registered in China Drug Clinical Trial Registration and Information Publicity Platform (http://www.chinadrugtrials.org.cn), the registration number was CTR20170876, and the full date of registration was 04/AUG/2017.

## Introduction

Peptic ulcer is a disease with significant morbidity and mortality worldwide [1]. Untimely treatment may lead to abdominal pain, gastrointestinal bleeding and gastric perforation [2, 3]. Omeprazole is proton pump inhibitor that is widely used to minimize morbidity and mortality the associated peptic ulcer [4, 5]. Given that omeprazole is an acid-labile compound, it is usually made into oral enteric-coated granules which can be absorbed from the small intestine within 3_6 hours [6]. In China, omeprazole enteric-coated capsules (AstraZeneca Pharmaceutical Co. Ltd.) were approved for marketing in 2013 by the National Medical Products Administration. However, no import or product registration application for the original AstraZeneca omeprazole enteric-coated capsules has been submitted yet. Therefore, there is no systematic study on the pharmacokinetics of omeprazole enteric-coated tablets in a Chinese population.

Large interindividual variations of omeprazole plasma concentrations have been found in several studies [7–9]. Many pharmacokinetic studies have been conducted to analyze the relationship between omeprazole level and its influencing factors, such as gender, age, weight and food, however, the results are controversial owing to the sample size limitations or the defects in experimental design, especially in a Chinese population. In addition, it is necessary to construct a plasma concentration prediction model of omeprazole for its responsible and safe use.

In this study, a pharmacokinetics study of omeprazole enteric-coated tablets was conducted and the effects of age and gender were explored in Chinese population. Moreover, a backpropagation artificial neural network (BPANN) model was developed to predict the plasma concentration of omeprazole.

## Methods

### Subjects

The study data were collected from 47 Chinese healthy subjects at the Second Affiliated Hospital of Zhejiang University, School of Medicine (Hangzhou, Zhejiang, China) in 2017. We enrolled male and female volunteers aged from 18 to 45 years and with a body mass index between 19 and 26 kg∙m^−2^ The inclusion criteria were as follows: (1) no clinically relevant abnormalities identified by subjects’ medical history, physical examination, clinical laboratory tests, vital signs, chest radiography, and 12-lead ECG; (2) no tobacco, drug, or alcohol abuse; (3) no breastfeeding, pregnancy or childbearing potential of female subjects during the study. The exclusion criteria were as follows: (1) positive blood screening results for HIV or hepatitis or any positive urine drug screen; (2) any hospital admission or major surgery, any donation of blood or acute loss of blood or any participation in other clinical trials within the previous 3 months; (3)no heavy tea or coffee consumption more than 1 L/day; (4) no history of allergies to the study medicines or related substances.

### Study design and safety assessment

A single dose of 20 mg omeprazole tablet (AstraZeneca Pharmaceutical Co. Ltd.) was administered with 240 mL of water after an overnight fast. A highly caloric meal was consumed within 30 min before drug administration and water was forbidden 1 h before and after drug administration. Blood samples (2 mL each) were collected in K_2_EDTA anticoagulant tubes at predose and at 0.5, 1, 1.5, 2, 2.5, 3, 3.5, 4, 4.5, 5, 6, 7, 8, 10, and 12 h postdose. The blood samples were centrifuged at 3000 g and stored at -80℃ until analysis. A validated liquid chromatography tandem mass spectrometry (LC–MS/MS) method was used to determine the plasma concentrations of omeprazole by Shanghai Xihua Scientific Co., Ltd.

For all studies, safety assessments included vital signs, 12-lead ECG, physical examinations, and clinical tests. Adverse events were evaluated with regard to their seriousness, intensity, time course, outcome, and relationship to the study drug.

### Pharmacokinetic statistical analysis

Pharmacokinetic analysis was performed by WinNonlin software (Version 6.4, Pharsight Corporation, Mountain View, California, USA), and a noncompartmental method was used to calculate pharmacokinetic parameters. A total of 47 subjects were divided into the low-age group (≤ 26 years, 24 subjects) and the high-age group (> 26 years, 23 subjects) based on the calculation of the median age. Pharmacokinetic data from the low-age and high-age groups or male and female groups were compared with a Student’s *t*-test.

### Principal component analysis

Demographic characteristics and routine biochemical and hematological parameters were collected from all of the subjects. A total of 12 variables were included and converted into independent or irrelative variables by principal component analysis (PCA). PCA is a mathematical algorithm that reduces the dimensionality of the data while retaining most of the variation in the data set. It accomplishes the reduction by identifying directions, called principal components, along which the variation in the data is maximal [10]. This approach can reduce the data dimension and maintain the most original variable information. The main calculation procedures of PCA were as follows: (1) the data collection from the subjects was conducted on standardized processing; (2) the characteristic value and feature vector of the correlation coefficient matrix R were calculated to define new indicator variables; (3) the principal components were chosen and the information contribution rate and accumulated contribution rate were calculated; (4) when the accumulated contribution was close to 1, we chose the principal components to replace the original variables and thereby obtain the key factors.

### Metaheuristic optimization algorithms

To get a better fitting effect of the BPANN model, we used particle swarm optimization algorithm (PSO), whale optimization algorithm (WOA), and genetic algorithm (GA) to optimize the model.

The PSO, presented by Eberhart and Kennedy in 1995, is a heuristic and evolutionary algorithm inspired by the behavior of birds to locate desirable positions in a given area through cooperation and competition [11]. Some entities, called particles, are scattered in the search space in the PSO [12]. The position of each particle represents a possible solution, and each solution is the way that in the search of a position in a space, particles change the flying distance and directions via changing the speed [13, 14]. Each particle remembers its optimal position *p*_*iD*_ in the searching history in the iteration process [15]. All of the optimal positions of all particles are the global optimal position *p*_*gD *_[16]. The equation and parameter of particle movement are as follows [17]:

$${V}_{iD}^{j+1}=\omega {V}_{iD}^{j}+{c}_{1}{r}_{1}\left({p}_{iD}^{j}-{x}_{iD}^{j}\right)+{c}_{2}{r}_{2}\left({p}_{gD}^{j}-{x}_{iD}^{j}\right)$$

$${x}_{iD}^{j+1}={x}_{iD}^{j}+{V}_{iD}^{j+1}$$

where $i$, $j$, $D$ stand for the particle, the current iteration amount and the particle dimension, respectively. ${x}_{iD}^{j}$ and ${V}_{iD}^{j}$ are the velocity and position in the $j$ iteration. Non-negative constant ${c}_{1}$ and ${c}_{2}$ are the learning factor, which determines the effects of ${p}_{iD}$ and ${p}_{gD}$ on the new velocity. ${r}_{1}$ and ${r}_{2}$ are the pseudo random amount evenly distributed in the interval [0, 1]. $\omega$ is the inertia weight, adjusting the searching ability in the solution domain [18, 19].

The WOA has been developed by inspiration from humpback whales that hunt by creating a bubble-net. The WOA takes place at three stages which are encircling, bubble-net attacking and searching for prey [20]. The equation and parameter of encircling are as follows:

$${X}_{k}^{j+1}={X}_{k}^{*}-{A}_{1}\bullet {D}_{k},$$

$${D}_{k}=\left|{C}_{1}\bullet {X}_{k}^{*}-{X}_{k}^{j}\right|,$$

$${C}_{1}=2{r}_{2}; {A}_{1}=2a {r}_{1}-a,$$

where ${X}_{k}^{*}$ stands for the best current location for whales; ${X}_{k}^{j+1}$ stands for the kth component of the spatial coordinate ${X}^{j+1}$; *a* is the coefficient in the iterative process (decreases linearly from 2 to 0 in the iterative process); ${r}_{1}$ and ${r}_{2}$ are random vectors between 0 and 1.

The equation and parameter of bubble-net attacking are as follows:

$${X}_{k}^{j+1}={X}_{k}^{*}+{D}_{k}\bullet {e}^{bl}\bullet \mathrm{cos}2\pi l,$$

$${D}_{k}=\left|{X}_{k}^{*}-{X}_{k}^{j}\right|,$$

where l is a random number in the interval of [-1, 1], and *b* is a constant for the formation of the spiral shape.

The final equation of searching for prey is as follows:

$${X}_{k}^{j+1}={X}_{k}^{rand}-{A}_{1}\bullet {D}_{k,}$$

$${D}_{k}=\left|{C}_{1}\bullet {X}_{k}^{rand}-{X}_{k}^{j}\right|,$$

$${C}_{1}=2{r}_{2}; {A}_{1}=2\mathrm{a}\cdot {r}_{1}-a,$$

The GA is a popular approach to achieve this optimization approach. The GA approach is inspired by the Darwin’s theory of natural selection survival of the fittest [21]. The equation of GA has been embedded in the MATLAB2020a.

### BPANN modeling

The BPANN is a kind of machine learning technology that minimizes the error between the network outputs and the desired outputs, adjusting the weights and biases by a small amount at a time through a gradient-based procedure [22, 23].The BPANN comprises two procedures: a forward stage where the input signals move forward through the network and a backward stage where the error is propagated backward from the output layer to the input layer. The error is calculated in the output layer and the parameters are updated for the direction in which the performance function most rapidly decreases [24].

**Fig. 1:**
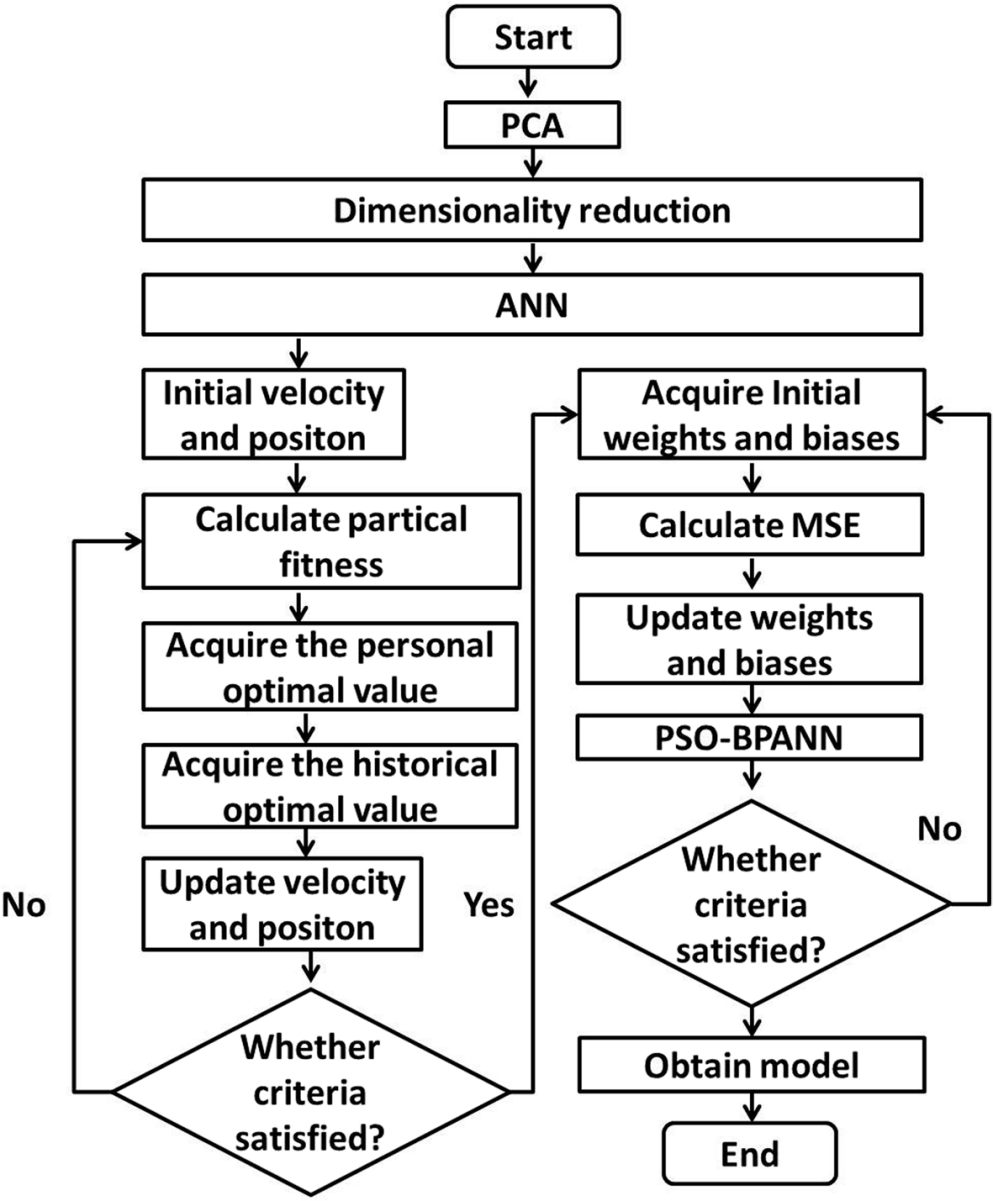
The overall calculation process of PCA and PSO-BPANN modeling

Although the BPANN algorithm is widely used, it might become stuck at the local minimum if the initial weights and biases are far from the optimal values that can give the global optimal solutions [25]. Several metaheuristic optimization algorithms, such as the PSO, GA, and harmony search algorithm, have been combined with the BPANN to overcome this shortcoming [25–27]. In this study, PSO was chosen to improve the performance of the BPANN owing to its simplicity and wide applicability. The variables selected were used as the input layer, and the plasma concentration of omeprazole was used as the output layer. The node numbers of hidden layer were determined based on the formula of l < n-1, where l was the number of the nodes in the hidden layer, and n was the number of nodes in the input layer, and then followed by the trial and error method to identify the best numbers of the node. Through the global search ability of the PSO algorithm, the initial weights and biases of the BPANN were obtained and the true global optimization and performance improvement were found. The overall calculation process is shown in Fig. 1.

## Results

### Subjects’ characteristics

A total of 47 subjects met the protocol criteria and were enrolled in the study. The characteristics of all subjects, low-age vs. high-age groups, and male vs. female groups are summarized in Table 1.

**Table 1:** The characteristics of all subjects, the low-age and high-age groups and male and female groups

Characteristics	All subjects	Male group	Female group	Low-age group	High-age group
Gender(male/female)	(23/24)	(23/0)	(0/24)	(13/11)	(10/13)
Age(years)	28.6 ± 6.61 (20.0,42.0)	27.6 ± 6.13 (21.0,42.0)	29.2 ± 6.95 (20.0,42.0)	23.3 ± 1.79 (20.0,26.0)	34.3 ± 4.68 (28.0,42.0)
Body mass index (kg∙cm-2)	27.2 ± 1.79 (19.2,25.3)	22.1 ± 1.89 (19.2,25.2)	21.4 ± 1.68 (19.3,25.3)	21.4 ± 1.96 (19.2,24.6)	22.2 ± 1.55 (19.3,25.3)
Hemoglobin count (g∙L-1)	141 ± 14.3 (117,172)	153 ± 7.78 (139,172)	130 ± 9.04 (117,149)	142 ± 13.6 (117,161)	140 ± 15.5 (117,172)
Red blood cell count (1012∙L-1)	4.69 ± 0.44 (3.77,5.52)	4.98 ± 0.28 (4.36,5.43)	4.43 ± 0.37 (3.77,5.52)	4.79 ± 0.40 (3.85,5.52)	4.59 ± 0.47 (3.77,5.43)
White blood cell count (109∙L-1)	5.84 ± 1.23 (4.20,9.10)	6.00 ± 1.17 (4.40,8.80)	5.70 ± 1.32 (4.20,9.10)	5.73 ± 1.38 (4.20,9.10)	5.93 ± 1.11 (4.20,7.90)
Blood platelet count (109∙L-1)	223 ± 52.9 (114,361)	216 ± 49.3 (114,307)	231 ± 57.4 (142,361)	215 ± 50.2 (148,361)	231 ± 56.6 (114,320)
Alanine aminotransferase (U∙L-1)	18.7 ± 3.37 (13.0,28.0)	19.0 ± 3.38 (14.0,26.0)	18.6 ± 3.46 (13.0,28.0)	18.8 ± 2.57 (14.0,25.0)	18.7 ± 4.11 (13.0,28.0)
Aspartate aminotransferase (U∙L-1)	14.7 ± 6.89 (6.00,38.0)	17.5 ± 7.25 (8.00,38.0)	11.9 ± 5.50 (6.00,28.0)	12.4 ± 5.02 (6.00,25.0)	14.1 ± 7.87 (7.00,38.0)
Blood urea nitrogen (mmol∙L-1)	4.14 ± 1.05 (1.30,7.00)	4.60 ± 0.94 (3.10,7.00)	3.69 ± 0.99 (1.30,5.80)	4.12 ± 0.97 (2.50,5.80)	4.17 ± 1.17 (1.30,7.00)
Serum creatinine (μmol∙L-1)	61.9 ± 12.7 (39.0,89.0)	72.3 ± 8.66 (54.0,89.0)	52.5 ± 5.80 (41.0,61.0)	65.1 ± 12.7 (46.0,89.0)	58.9 ± 12.5 (39.0,79.0)

Data are shown as Mean ± SD (min, max)

#### Pharmacokinetics

The area under the concentration–time curve from dose to last measurable concentration (AUC_0-t_), the area under the concentration–time curve from dose to infinity (AUC_0-∞_), the maximum plasma concentration (C_max_), the time from dose to C_max_ (T_max_), and the half-life of terminal elimination (t_1/2_) were calculated. The results of the pharmacokinetic parameters of all subjects, low-age vs. high-age groups, and male vs. female groups are summarized in Table 2.

**Table 2:** The pharmacokinetics parameters of all subjects, the low-age and high-age groups and male and female groups

Pharmacokinetics Parameters	All subjects	Male group	Female group	Low-age group	High-age group
AUC0-t(ng∙h∙mL-1)	980 ± 1121	1067 ± 1232	715 ± 1000	1262 ± 1410	496 ± 486
AUC0-∞(ng∙h∙mL-1)	1063 ± 1264	1144 ± 1384	782 ± 1138	1393 ± 1598	506 ± 499
Cmax(ng∙mL-1)	379 ± 297	425 ± 341	283 ± 234	435 ± 330	266 ± 234
Tmax(h)	4.50(1.00,8.00)	4.00(1.00,5.50)	4.50(1.50,8.00)	4.50(1.50,8.00)	4.50(1.00,6.00)
t1/2(h)	1.25 ± 0.64	1.27 ± 0.71	1.14 ± 0.59	1.44 ± 0.81	0.97 ± 0.27

AUC0-t: Area under the concentration–time curve from dose to last measurable concentration

AUC0-∞: Area under the concentration–time curve from dose to infinity

*Cmax* Peak concentration, *Tmax* Time from dose to Cmax, *t1/2* Half-life of terminal elimination

Data are shown as Mean ± SD except for Tmax which are shown as median (range)

### Effects of age and gender

**Fig. 2:**
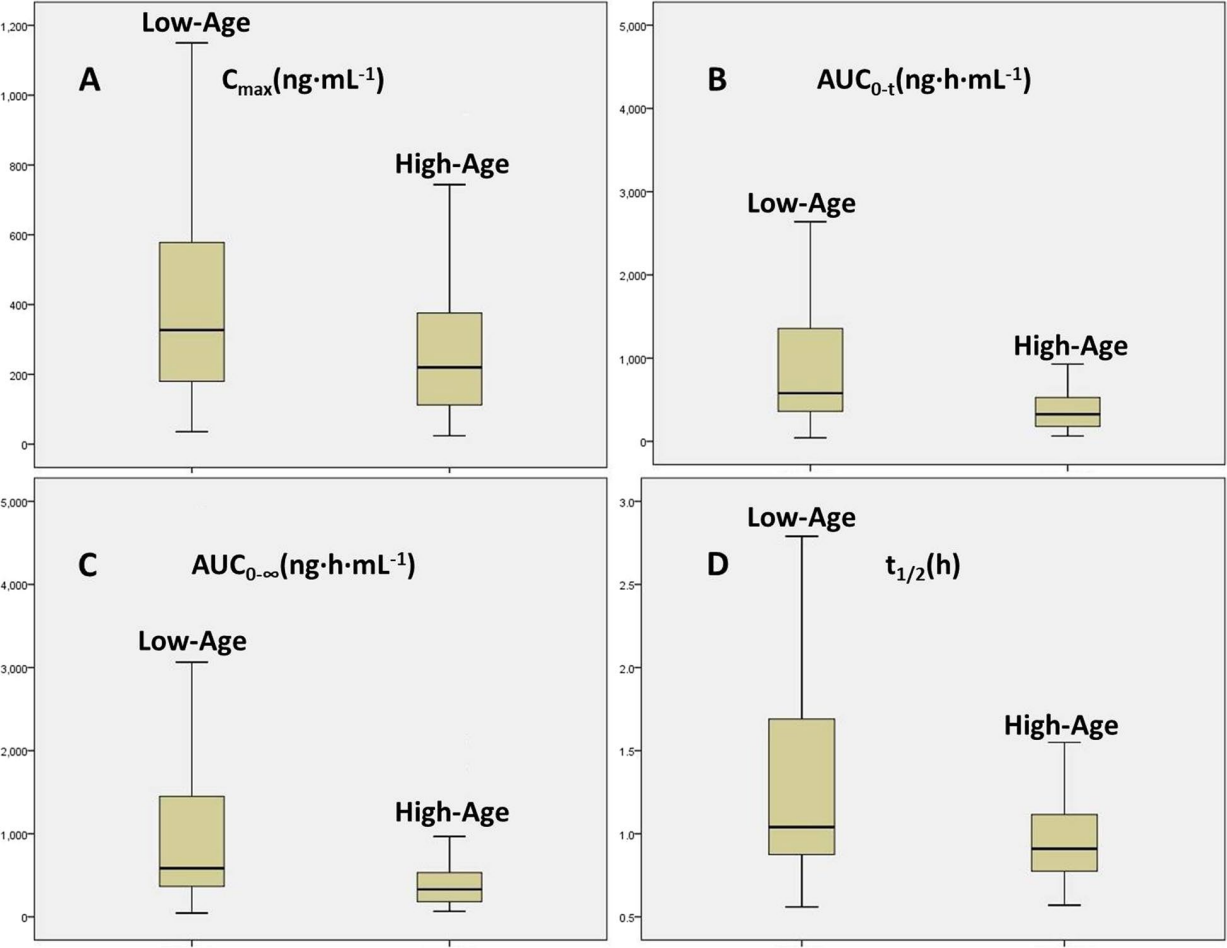
The peak plasma concentration (Cmax; **A**), area under the pharmacokinetic time curve (AUC0-t; **B** and AUC0-∞; **C**) and half-life of terminal elimination (t1/2; **D**) for low-age and high-age groups following oral administration of omeprazole under the fed state. *P* < .005

The effects of age and gender were analyzed using a Student’s *t*-test in SPSS Statistics 19, and the results’s shown in Table 3. We found that the C_max_, AUC_0-t_, AUC_0-∞_ and t_1/2_ values significantly differed between the low-age and the high-age groups (shown in Fig. 2). However, there were slight or no significant differences in the pharmacokinetic parameters between male and female subjects.

**Table 3:** The effect of pharmacokinetic parameters of Age and Gender in healthy Chinese subjects

Pharmacokinetics Parameters P	Age	Gender
Cmax(ng∙mL-1)	< .05(.049)	.103
Tmax(h)	.921	.210
AUC0-t(ng∙h∙mL-1)	< .05(.017)	.287
AUC0-∞(ng∙h∙mL-1)	< .05(.014)	.233
t1/2(h)	< .05(.011)	.514

AUC0-t: Area under the concentration–time curve from dose to last measurable concentration

AUC0-∞: Area under the concentration–time curve from dose to infinity

*Cmax* Peak concentration, *Tmax* Time from dose to Cmax, *t1/2* Half-life of terminal elimination

### PCA results

The PCA results of 12 variables collected from Chinese healthy subjects are shown in Table 4. The largest seven characteristic values in the matrix were 3.693, 2.252, 1.220, 1.184, 1.000, 0.869, and 0.659, and all of them were over 0, and the comprehensive information contribution rate was 90.648%. Hence, the seven principal components basically maintained the original information of all of the indicators, and were able to fully reflect the changing trend. Thus, it was feasible and valid to choose the 7 principal components to replace the original 12 indicators.

**Table 4:** Characteristic values, contribution rate and accumulated contribution rate of PCA

Variable	Characteristic value	Contribution rate	Accumulated contribution rate
× 1	3.693	30.774	30.774
× 2	2.252	18.771	49.545
× 3	1.220	10.165	59.710
× 4	1.184	9.869	69.579
× 5	1.000	8.333	77.912
× 6	0.869	7.242	85.154
× 7	0.659	5.494	90.648
× 8	0.354	2.946	93.594
× 9	0.281	2.341	95.935
× 10	0.242	2.018	97.952
× 11	0.155	1.296	99.248
× 12	0.090	0.752	100.000

### Metaheuristic optimization algorithms results

Table 5 shows the mean model prediction results of the BPANN for the plasma concentration. When the BPANN model prediction was examined, the PSO achieved the best result compared with the other two algorithms. For this reason, the PSO-BPANN was used in the prediction of the plasma concentration of omeprazole in human.

**Table 5:** The comparison of prediction results for PSO, WOA and GA model

	R2	MAE	RMSE
PSO	0.932	0.080	0.133
WOA	0.832	0.150	0.209
GA	0.854	0.131	0.195

### PSO-BPANN result

**Fig. 3:**
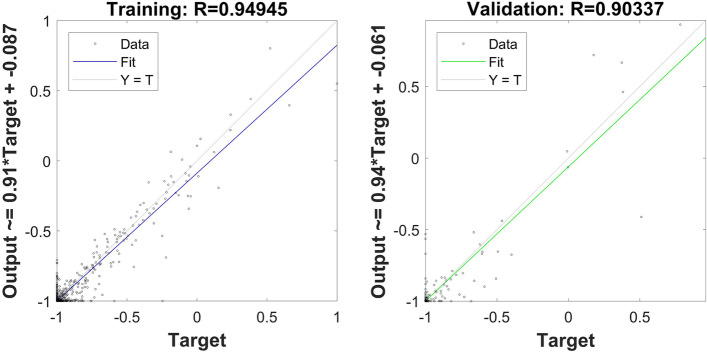
The correlation between measured and predicted plasma concentration of omeprazole by BPANN in the training and validation groups

**Fig. 4:**
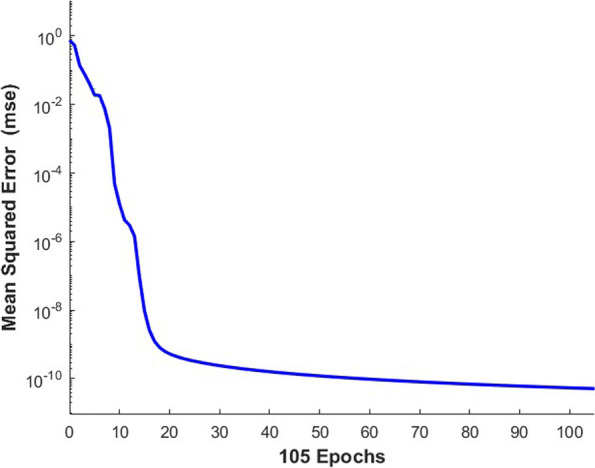
The PSO-BPANN converge curve

According to the results of PCA, the final established BPANN model consisted of one input layer with 7 neurons, 1 hidden layer with 13 nodes, and 1 output layer with one node (plasma concentration of omeprazole). MATLAB2020a was used as the processor. All the data (847 data points) collected were randomly divided into a training group (70%, 591 data points), a validation group (15%, 128 data points), and a test group (15%, 128 data points) which involved in none process of modeling. To eliminate the effects of input variables, Mapminmax function was used to normalize the sample input data into [-1, 1]. PSO was used to optimize the initial weight and threshold to acquire the optimal parameters and the parameters of the PSO were set as follows: population size, 50; evolutionary generation, 100; acceleration factor *c*_*1*_ = *c*_2_ = 1.49445; intervals of particle position and velocity [-5, 5] and [-1, 1], respectively. Many hyperparameters were used to train the net, where the number of training iterations was set to 1000, the network performance target was 10^−7^, and the learning rate was 0.001. The transfer function for the hidden layer was “logsig”, and that for the output layer was “purelin.” The Levenberg–Marquardt optimization method “trainlm” was the training function, and “leanngdm” was the threshold learning function. After the model was trained, the performance of the network was evaluated by the following four metrics: the MSE, magnitude of the gradient, number of validation checks, and correlation coefficient. The results indicated good performance: 0.000355 for MSE, 0.000133 for the magnitude of the gradient, and 50 for the number of validation checks. The correlation coefficient of training and validation are shown in Fig. 3 and the PSO-BPANN converge curve is shown in Fig. 4.

### Application and MIV result

**Fig. 5:**
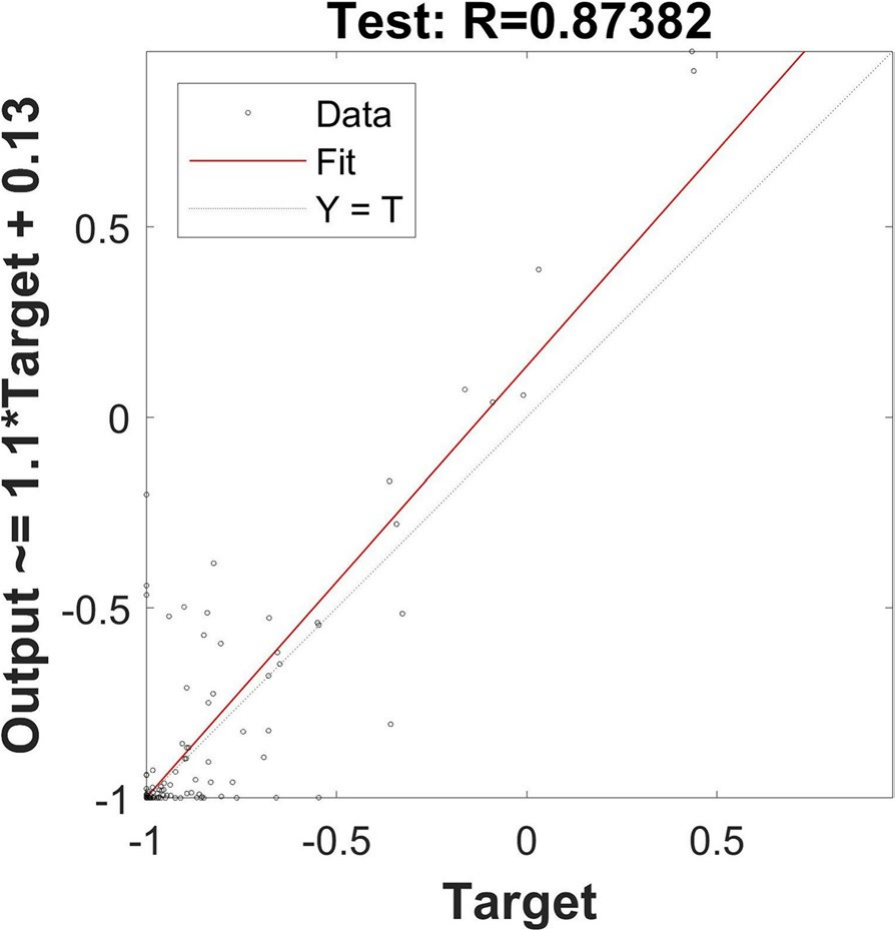
The correlation between measured and predicted plasma concentration of omeprazole by BPANN in the test groups

The constructed PSO-BPANN model was used to predict the plasma concentration of omeprazole in the test group. The results of the predicted values compared with the measured values are shown in Fig. 5, and the correlation coefficient was calculated. The data collected from predicted and measured values were analyzed with a paired-samples *t*-test (shown in Table 6). All the results of correlation coefficient (*R* = 0.874) and paired-samples t test (*P* > 0.05) showed good fitness of the PSO-BPANN model.

**Table 6:** The result of paired-samples t test of predicted and measured values

	Mean Value	Standard Error of Mean	t	P
Predicted values	105	19.7	1.352	.179
Measured values	91.8	15.2		

The mean impact value (MIV) is an indicator that represents the magnitude of the effect of the input value on the output value. The calculation process of MIVs was as follows: (1) new training samples were formed by increasing and decreasing 10% of each input variable values; (2) new training samples were incorporated into the PSO-BPANNs, and the two results were subtracted and divided by the number of observations; (3) the sign of the MIV results represented the positive and negative correlation between the variable and the result, and the absolute values of the MIV results represent the importance of the variables. The results of MIVs are shown in Table 7.

**Table 7:** The value of the mean impact value (MIV) of 12 variables

Covariate	Mean effect value	Covariate	Mean effect value
Body mass index	95.13	White blood cell count	-18.83
Blood urea nitrogen	31.10	Hemoglobin count	-20.52
Red blood cell count	25.19	Aspartate aminotransferase	-31.49
Alanine aminotransferase	25.11	Serum creatinine	-105.55
Blood platelet count	18.22	Gender	-127.56
Time	5.19	Age	-150.14

## Discussion

In this study, we analyzed the effects of age and gender on the plasma concentration of omeprazole in a Chinese population. Moreover, the PCA and PSO-BPANN algorithms were used to predict the blood concentration of omeprazole for the first time.

To our knowledge, this is the first time that the effects of age and gender and the pharmacokinetics parameters of omeprazole enteric-coated tablets were explored in a Chinese population. Considering that age and gender are usually considered key factors that could alter the pharmacokinetic profiles of drugs, leading to variation in systemic exposure, we introduced their effects into the omeprazole pharmacokinetics study in healthy subjects. The results showed the C_max_ was reduced by 38.8% in the high-age group compared with the low-age group. Meanwhile, the mean systemic exposure was 60.7% decreased (average of AUC_0-t_) in the high-age group. In our study, the effect of age was found obvious (*P* < 0.05) in the Chinese population and might contribute to the lower metabolic capacity of the high-age group. In addition, although there were no obvious differences (*P* < 0.05) between males and females in our study, the reduction amount of C_max_ and AUC_0-t_ were 33.4% and 33.0%, respectively, in the female group, compared with the male group. It is necessary to understand the age and gender effects on omeprazole, which might improve management of patients with peptic ulcer and minimize the associated morbidity and mortality.

BPANN was widely used during our pharmacokinetic research and plasma concentration prediction. In previous research, we predicted the plasma concentration and pharmacokinetic parameters of four bioequivalence studies of rosuvastatin calcium tablets [28], predicted pharmacokinetics and the effect of genetic polymorphisms of deferasirox [29], and predicted the plasma concentration of febuxostat from different formulations [30]. Although we eventually achieved good prediction results, the stuck at the local minimum of the BPANN and the variable selection introduced were significant challenges during the research process. In this study, the PCA and PSO were introduced into modeling and the final model PSO-BPANN became more reliable and stable. Moreover, the MIV of gender and age was -127.56 and -150.14, respectively, which indicated strong negative effects on the plasma concentration of omeprazole. The results of the MIV are consistent with our previous conclusion. In addition, the MIV of body mass index and serum creatinine was 95.13 and -105.55, respectively. We suggest that the positive effect of body mass index and the negative effect of serum creatinine should not been ignored, either.

Although the low number of subjects included in the study did not allow us to explore the effect of more variables on the pharmacokinetics of omeprazole enteric-coated tablets, such as the CYP genetic polymorphisms [31], the model we established still had a good predictive effect on the plasma concentration of omeprazole in the Chinese population. We plan to introduce the effect of genetic polymorphisms into the PSO-BPANN in subsequent studies.

## Conclusions

In this study, the effects of age and gender on pharmacokinetics study of omeprazole enteric-coated tablets in Chinese population were explored and a PSO-BPANN model was developed to predict the plasma concentration of omeprazole.

## Data Availability

All data and materials included in this study are available upon request by contact with the corresponding author.
